# Motorized Intramedullary Bone Transport Nail for Reconstruction of a Large Diaphyseal Bone Defect after Tumor Resection in a Child—A Case Report

**DOI:** 10.3390/children13010026

**Published:** 2025-12-23

**Authors:** Farah Selman, Valentine Schneebeli, Stijn de Joode, Daniel Müller, Thomas Dreher

**Affiliations:** 1 Department of Orthopedic Surgery, Balgrist University Hospital, University of Zurich, Forchstrasse 340, 8008 Zürich, Switzerland; farah.selman@balgrist.ch (F.S.); sdejoode55@gmail.com (S.d.J.); daniel.mueller@balgrist.ch (D.M.); or thomas.dreher@kispi.uzh.ch (T.D.); 2 Department of Orthopedic Surgery and Traumatology, Zuyderland Medical Center, 6162 Sittard-Geleen, The Netherlands; 3 Care and Public Health Research Institute (CAPHRI), Faculty of Health, Medicine and Life Sciences, 6200 Maastricht, The Netherlands; 4 Department of Pediatric Orthopedics and Traumatology, University Children’s Hospital, University of Zürich, Steinwiesstrasse 75, 8032 Zürich, Switzerland

**Keywords:** osteofibrous dysplasia-like adamantinoma, intramedullary bone transport nail, tumor resection, reconstruction

## Abstract

**Highlights:**

**What are the main findings?**

- The motorized Intramedullary Bone Transport Nail (IMBTN) successfully reconstructed a large tibial defect in a pediatric patient after tumor resection.
- Skeletal growth and alignment were preserved without tumor recurrence.

**What are the implications of the main findings?**

- IMBTN provides an internal, less invasive alternative to traditional reconstructive methods in pediatric oncologic limb reconstruction.
- IMBTN enables controlled bone transport without disrupting skeletal growth in immature patients.

**Abstract:**

Background: Reconstructing large bone defects in pediatric patients after tumor resection is challenging, as conventional techniques are associated with high complication rates and morbidity. The Intramedullary Bone Transport Nail (IMBTN) may reduce these complications while preserving limb alignment and skeletal growth in pediatric oncologic reconstruction. Methods: A 15-year-old female with an osteofibrous dysplasia-like adamantinoma of the tibial diaphysis underwent complete en-bloc resection, leaving a 9 cm bone defect. An IMBTN (Precice, NuVasive) was implanted for distraction osteogenesis, with distraction starting eight days post-surgery at 0.25 mm twice daily. Follow-up visits monitored bone healing, alignment, and limb length. Results: The 9 cm defect was successfully reconstructed, with complete bone healing at the distraction site. Complete consolidation was confirmed at 18 months. The transport nail was removed at two years, and no further revisions were necessary. At two-year follow-up, the patient reported minimal pain on the Visual Analog Scale 1/10, and no recurrence of the tumor was noted. Conclusions: The use of IMBTN for large bone defect reconstruction following tumor resection in pediatric patients is a safe and effective technique. It enables stable bone transport while preserving alignment, maintaining limb length, and is less invasive than traditional reconstructive approaches.

## 1. Introduction

Reconstruction of large diaphyseal bone defects following tumor resection in skeletally immature patients remains one of the most demanding challenges in pediatric orthopedic oncology. Beyond restoring structural continuity and mechanical stability, surgeons must preserve physeal-mediated longitudinal growth, maintain proper limb alignment, prevent deformities, and minimize long-term morbidity.

Historically, distraction osteogenesis using external fixation, introduced by Ilizarov in the 1950s [1,2,3,4], has been used for segmental bone reconstruction, but it is associated with complications such as pin-tract infections and joint contractures [3,5,6,7]. Alternative biologic strategies, including vascularized bone autografts [8] and induced membrane techniques [9], provide structural and biological support for bone healing, but carry risks of donor-site morbidity, prolonged treatment duration, non-union, or graft resorption [10,11,12].

Magnetically controlled intramedullary bone transport nails, including the Precice Intramedullary Bone Transport Nail (IMBTN), represent a novel internal strategy that enables precise, frame-free distraction osteogenesis while potentially reducing the complications associated with traditional reconstructive methods [13,14,15,16]. While widely used in adults for limb lengthening and reconstruction after trauma or tumor resection, their application in pediatric patients following tumor resection remains scarce.

This case report aims to demonstrate the feasibility of IMBTN for large bone defect reconstruction following pediatric tumor resection.

## 2. Case Presentation

**Figure 1:**
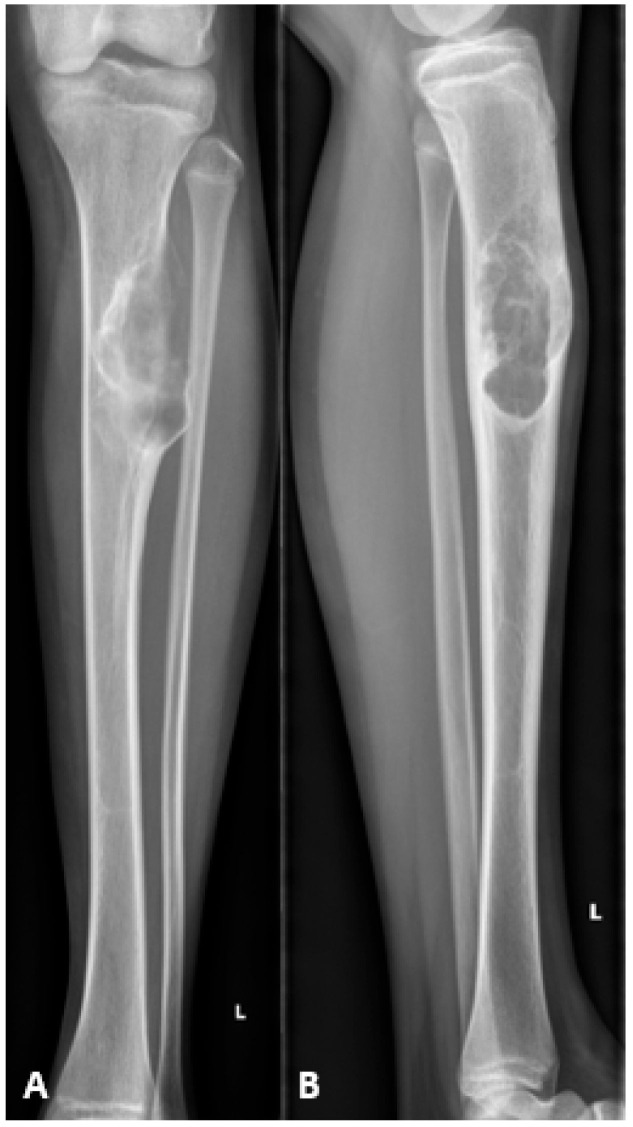
Preoperative radiographs showing an osteofibrous dysplasia-like adamantinoma (OFD/LA) in the left tibial diaphysis. (**A**) Anteroposterior view. (**B**) Lateral view.

**Figure 2:**
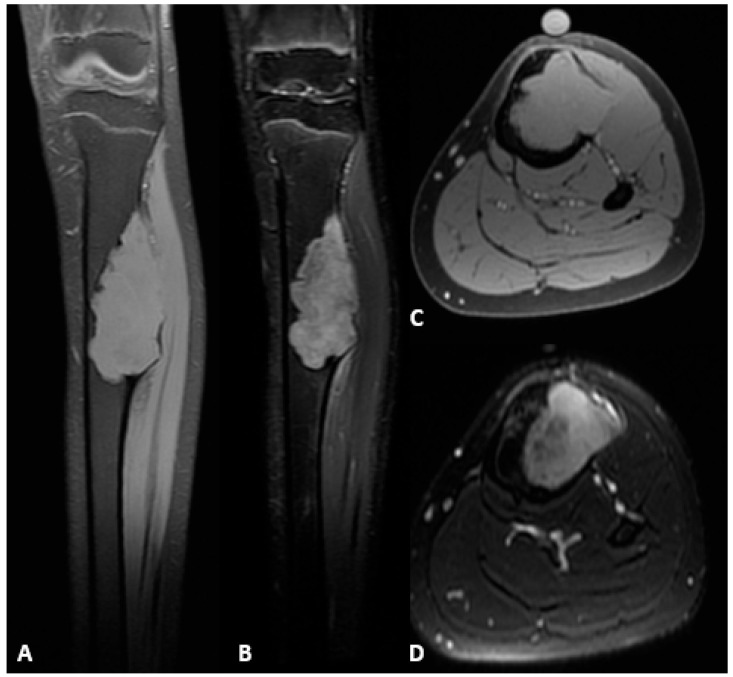
Preoperative MRI of the left tibial diaphysis demonstrating an OFD/LA. (**A**) Hypointense lesion on T1-weighted sequence, coronal plane. (**B**) Hyperintense lesion on T2-weighted fat-suppressed sequence, coronal plane. (**C**) Hypointense lesion on T1-weighted sequence, axial plane. (**D**) Hypointense lesion on T2-weighted sequence, axial plane.

A 15-year-old female, skeletally immature (162 cm tall, 41.1 kg, BMI 15.6 kg/m^2^, Risser grade 3), presented with a 6-month history of moderate, atraumatic pain in the left proximal tibia, worsened by walking and limiting daily activities. Clinical examination revealed an antalgic gait and a palpable, warm swelling of the proximal tibia with pain on ankle dorsiflexion. Imaging studies, including MRI and X-ray, revealed a lesion in the proximal tibia measuring 7.6 × 3.0 × 3.6 cm. (Figure 1 and Figure 2). Histopathological analysis demonstrated an osteofibrous dysplasia-like adamantinoma (OFD/LA), a rare variant of adamantinoma characterized by a fibro-osseous stroma containing interspersed epithelial nests. Although OFD/LA shares several characteristics with conventional adamantinoma, it exhibits lower aggressiveness and reduced metastatic potential. Despite these features, the lesion’s considerable volume, cortical breach, and high risk of pathological fracture necessitated a radical en-bloc resection with histologically confirmed margins.

## 3. Surgical Treatment

**Figure 3:**
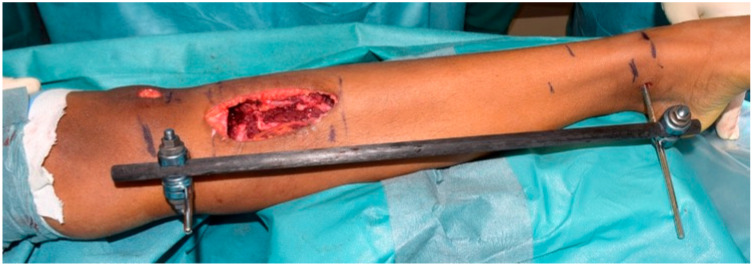
External fixator with carbon rod demonstrating the tibial defect following tumor resection.

**Figure 4:**
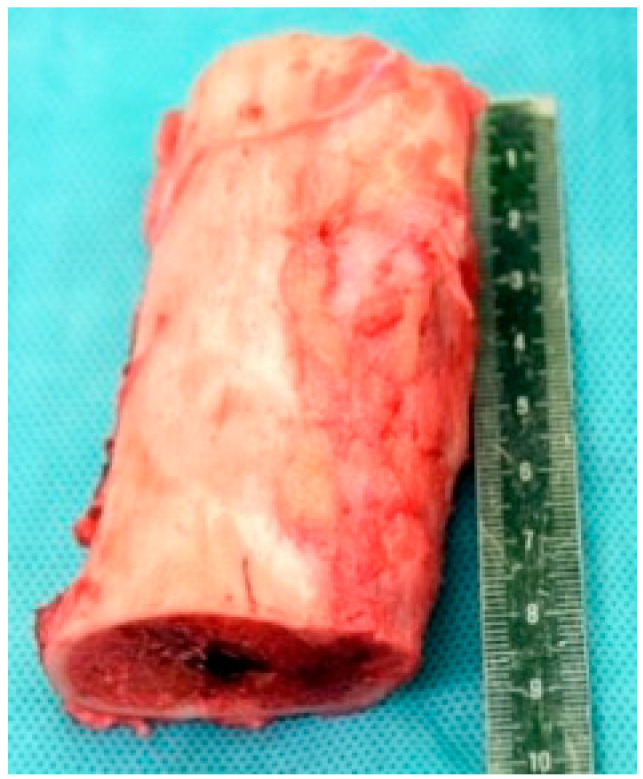
Resected tibial segment demonstrating an OFD/LA.

**Figure 5:**
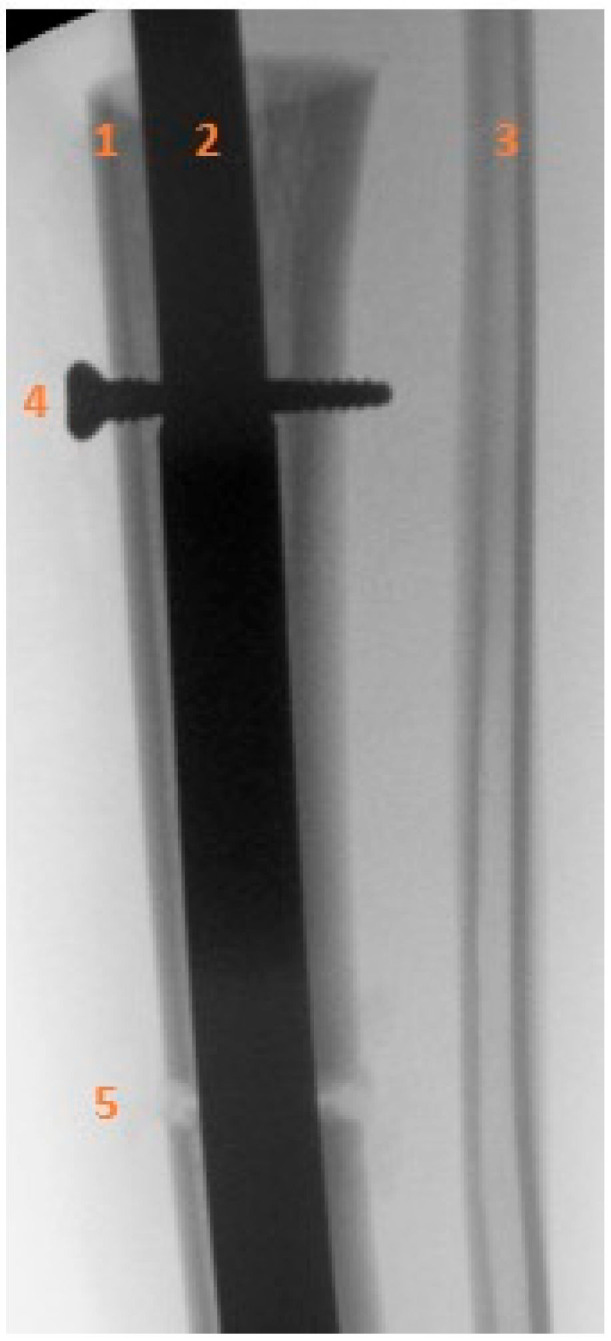
Intraoperative anteroposterior radiograph showing the locking bolt inserted into the transport segment. (1) Proximal tibial diaphysis and transport segment. (2) Intramedullary nail. (3) Intact fibular diaphysis. (4) Locking bolt within the transport segment and internal motor of the intramedullary nail. (5) Distal tibia, osteotomy of the transport segment.

The surgical procedure was performed under general anesthesia with the patient in a supine position. A standard midline infrapatellar incision was made to access the tibia. A Kirschner wire was positioned under fluoroscopic control to guide the procedure. The surgical sequence was as follows:

1. Placement of Steinmann pins and external fixator: Two bicortical Steinmann pins were inserted medially in the proximal and distal tibial segments and connected with a carbon rod external fixator to maintain alignment and rotational control before tumor resection (Figure 3).
2. En-bloc tumor resection: The tibial tumor was excised with a 1-cm margin, resulting in a 9-cm diaphyseal defect. The specimen was sent for histological analysis (Figure 4).
3. Insertion of the bone transport nail: A 360° Precice Bone Transport Nail (11 mm diameter, NuVasive Specialized Orthopedics, Aliso Viejo, CA, USA) was inserted through an entry point in the proximal metaphysis, just below the growth plate, maintaining a safe distance of at least 2 mm from the proximal tibial physis to avoid affecting the growth plate. The Steinmann pins and external fixator were maintained during nail insertion to preserve alignment.
4. Distal percutaneous osteotomy: A safe diaphyseal osteotomy was performed at a distance from the distal physis, using an oscillating saw under fluoroscopic guidance.
5. Fixation of the transport segment: A locking bolt was inserted into the transport segment to engage the internal transport mechanism of the intramedullary nail (Figure 5).
6. Nail locking: The nail was locked proximally and distally to achieve stable fixation.
7. Removal of temporary fixation: Once nail fixation was confirmed, the Steinmann pins and external fixator were removed.

## 4. Postoperative Follow-Up and Outcomes

Postoperative pain was initially managed with a sciatic catheter for four days. Upon removal, analgesia was maintained with opioid reserves as needed. At discharge on postoperative day 8, pain was controlled with paracetamol and non-steroidal anti-inflammatory drugs.

Physiotherapy was initiated immediately with early knee and ankle flexion and extension exercises and progressive muscle strengthening. Partial weight-bearing of 20 kg was allowed from two weeks postoperatively with progression to full weight-bearing guided by patient tolerance and radioclinical evaluation confirming hardware integrity without evidence of nail bending, screw migration, or shortening of the motorized implant.

**Figure 6:**
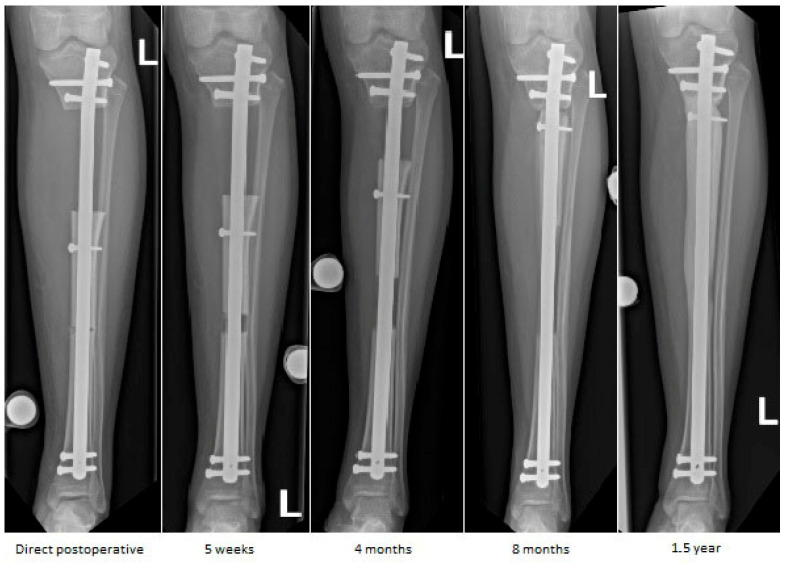
Sequential anteroposterior radiographs following retrograde tibial bone transport at direct postoperative, 5 weeks, 4 months, 8 months, and 1.5 years. The initial segmental defect measured 9 cm. Remaining defect measured 8.5 cm at 5 weeks, 7 cm at 4 months, 1.5 cm at 8 months, and fully consolidated at 1.5 years. Progressive transport and callus formation are evident, with complete restoration of tibial length and alignment at 1.5 years. Distances are approximate due to image scaling.

Distraction osteogenesis was initiated on postoperative day 8, one day later than the standard day 7, as a precaution to optimize early bone regenerate formation and minimize the risk of growth plate disturbances in this skeletally immature patient. The initial distraction rate was 0.5 mm/day (0.25 mm twice daily) and was gradually reduced toward the end to 0.25 mm/day to allow precise docking. Distraction continued for 230 days, achieving a transport distance of 8.5 cm, corresponding to near-complete defect closure. During distraction phase, follow-up visits were conducted every two weeks and included both clinical and radiographic evaluation to monitor bone transport and regenerate consolidation (Figure 6). Radiographic evaluation included annual MRI to surveil for local tumor recurrence. Clinical evaluation included monitoring for pain, joint range of motion, gait quality, muscle function, and neurovascular status, allowing for the early detection of complications such as joint contracture, stiffness, or muscle weakness.

At six months postoperatively, a transport screw exchange was performed after the patient reported audible blocking during distraction using the external magnetic controller. Radiographs demonstrated stable positioning of the intramedullary nail and locking screws, without secondary displacement. The symptoms were interpreted as being related to functional blocking of the transport screw. The screw was therefore exchanged and repositioned slightly proximal to its initial position to allow continued, controlled advancement of the transport segment along the intramedullary nail.

**Figure 7:**
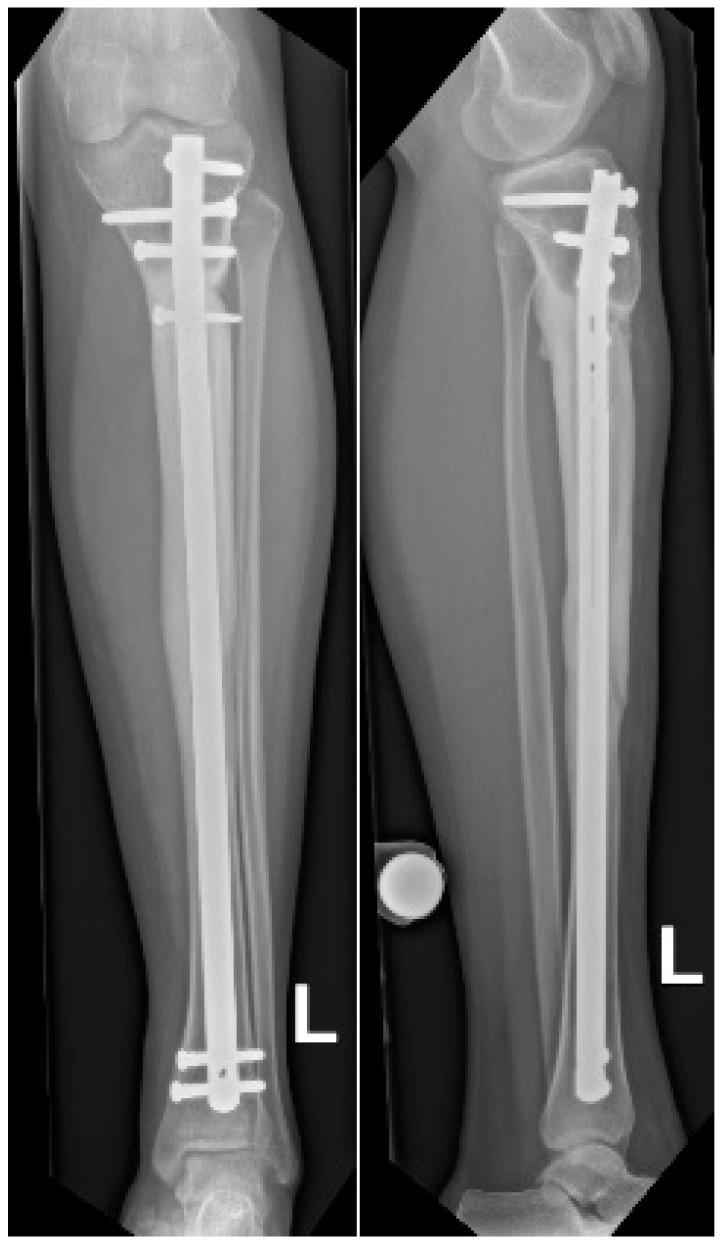
Radiographs at 18 months follow-up showing anteroposterior view (**left**) and lateral view (**right**) of the tibia. Consolidation can be observed, demonstrated by bridging callus across cortices and the absence of visible fracture lines, with restoration of tibial alignment.

**Figure 8:**
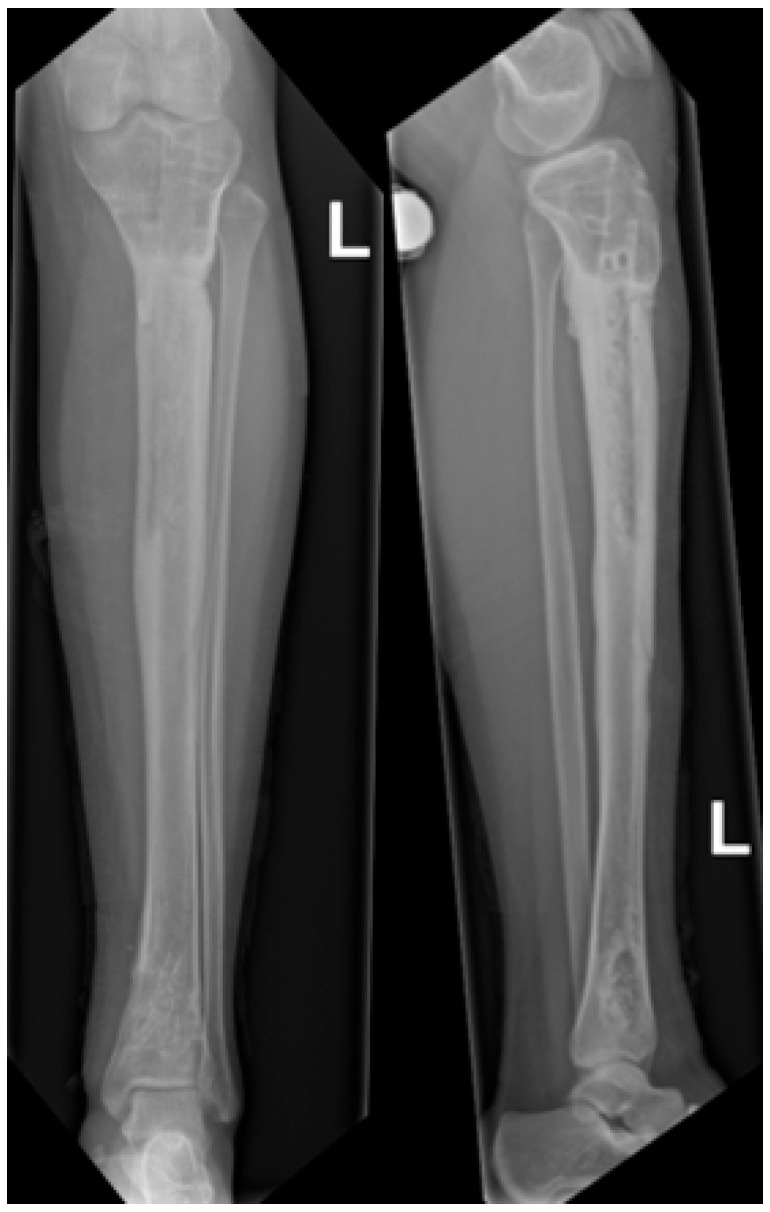
Radiographs following hardware removal, showing anteroposterior view (**left**) and lateral view (**right**) of the tibia. Slight procurvatum and varus alignment are noted.

At 18 months postoperatively, radiographs confirmed complete consolidation at both the docking and distraction sites (Figure 7). At two years postoperatively, complete hardware removal was performed (Figure 8).

**Figure 9:**
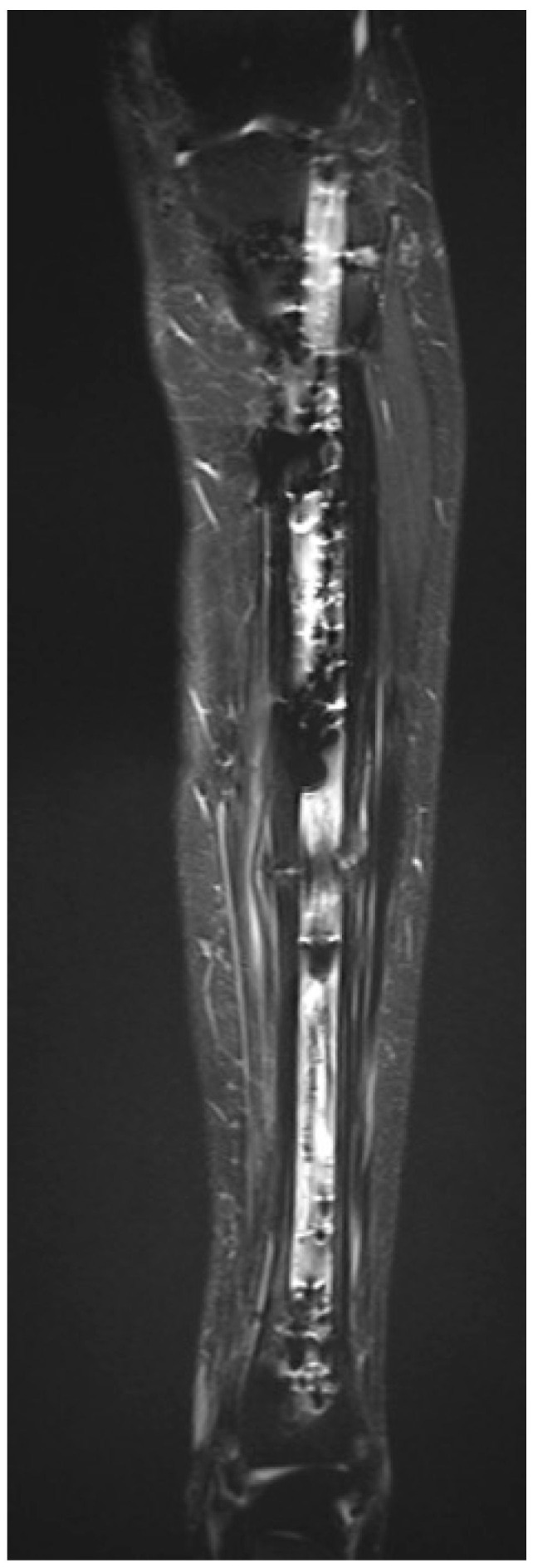
Postoperative MRI following hardware removal, T1 TIRM sequence, coronal plane, showing no residual lesion, no abnormal signal, and preserved tibial architecture at the previous lesion site, consistent with absence of recurrence.

At two-year follow-up, the patient showed marked improvement compared to preoperative status, reporting only occasional mild pain related to weather changes on the Visual Analog Scale 1/10, with no functional limitations. Clinical examination revealed a normal gait, healed and non-tender surgical scars, full range of motion of the knee and ankle, and preserved motor function. Residual hypoesthesia over the lateral lower leg was present without functional impact. Based on clinical assessment and patient-reported function, a retrospective Musculoskeletal Tumor Society score was estimated at 28/30. The patient reported normal quality of life and unrestricted participation in daily activities. No limb-length discrepancy or coronal/sagittal deformity was observed. Postoperative MRI showed no recurrence of the tumor (Figure 9).

## 5. Discussion

### 5.1. Main Findings

This case presents the successful reconstruction of a 9 cm tibial defect following tumor resection in a skeletally immature patient, with complete healing, no malalignment, and no recurrence at 2 years. The patient in this case presented with OFD-AD, a rare, locally aggressive bone tumor recently reclassified by the WHO as intermediate [17], distinct from conventional low-grade adamantinoma. Standard treatment is wide surgical resection with clear margins, as the tumor is resistant to chemotherapy and radiotherapy [18,19]. Reconstruction typically involves bone grafting (autograft, allograft, or vascularized fibula) or segmental techniques depending on defect size [20,21,22]. To date, the use of an intramedullary nail for OFD-AD has not been reported.

### 5.2. Alternative Treatments

Traditional methods for large tibial defects after tumor resection include external fixators, typically indicated in younger children with open physes, small medullary canals, or complex deformities [23,24], but are associated with pin-tract infections and functional limitations [3,5,6,7]. Alternative techniques such as distraction epiphysiolysis or Capanna’s method [25] provide structural stability and biological osteogenesis but carry risks of donor-site morbidity, delayed or non-union, allograft fracture, and joint stiffness, and are generally reserved for patients near skeletal maturity [10,26,27].

### 5.3. Feasibility of IMTBN

The successful application of the IMTBN, a magnetically controlled system, demonstrates its feasibility for pediatric oncologic reconstruction. This technique is specifically indicated for tibial lengthening in skeletally immature patients who are near skeletal maturity, have sufficient medullary canal diameter, minimal remaining growth, and no open physes at the planned entry site [28,29]. The feasibility of tibial bone transport using a motorized nail was first demonstrated by Kold et al. [30] in adults, showing successful reconstruction with fewer complications compared with external fixation. Accadbled et al. [31] reported a series of adolescent patients undergoing post-tumor reconstruction with a motorized nail, demonstrating low complication rates and satisfactory bone healing. However, this technique has several challenges.

### 5.4. Technical Challenges and Planning

Meticulous preoperative planning and intraoperative precision are essential to ensure correct alignment of the transport segments. Technical challenges include limited control over metaphyseal segments of limited length and potential structural weakness at the nail slot, which could compromise long-term stability [32,33,34].

### 5.5. Biological Considerations

Biological considerations, such as preservation of the endosteal blood supply, are crucial when selecting nail diameter, as larger sizes can interfere with bone healing and regenerate formation [35]. Controlled distraction rates, early mobilization, and close clinical monitoring ensure optimal regenerate formation and mechanical stability.

### 5.6. Complications from the Literature and Comparison to This Case

Reported complications in the literature include major adverse events in approximately 25% of IMTBN cases, predominantly malalignment and non-union [13,36]. In addition, pediatric use of elastic stable intramedullary nails for extensive osteofibrous dysplasia lesions has been associated with up to 50% anterior tibial angular deformity, particularly in circumferential lesions [37]. In this case, none of these complications occurred, likely due to meticulous preoperative planning with careful osteotomy avoiding the physis, precise intraoperative technique under radiologic control using Steinmann pins for alignment and rotational control, and close postoperative monitoring including early physiotherapy with progressive range of motion and weight-bearing.

### 5.7. Study Limitations

However, this study has some limitations. First, this is a single-patient report with short follow-up of 2 years. OFD-AD is known for late recurrences, and a 2-year follow-up may not capture all cases of local recurrence [38,39]. Other limitations include a lack of formal functional outcome scores, limited cost data, the learning curve associated with IMTBN placement and system availability as well as cost considerations. Future studies involving larger cohorts and longer-term surveillance are warranted to assess durability, functional outcomes, and reproducibility, and to further define the role of IMTBN in pediatric oncologic reconstruction.

## 6. Conclusions

The use of a motorized Intramedullary Bone Transport Nail may represent a promising strategy for reconstructing large segmental bone defects following tumor resection in pediatric patients. In this case, the fully internal, magnetically controlled system enabled stable bone transport while preserving alignment, maintaining limb length, and was less invasive than traditional reconstructive approaches. These findings highlight its potential value in pediatric oncologic reconstruction.

## Data Availability

The original contributions presented in this study are included in the article. Further inquiries can be directed to the corresponding authors.

## Abbreviations

The following abbreviations are used in this manuscript:

OFD/LA	Osteofibrous dysplasia-like adamantinoma
IMBTN	Intramedullary Bone Transport Nail
